# Supplementary material for: Applying network analysis to assess the development and sustainability of multi-sector coalitions
Source: PLoS One. 2022 Oct 18;17(10):e0276114. doi: 10.1371/journal.pone.0276114 (PMC9578585; doi:10.1371/journal.pone.0276114)
Supplement: S2 File — Survey distributed via email to ACH survey sample. (PDF) [file pone.0276114.s002.pdf]

Addendum A: ACH Network Survey

While each site administered a slightly different survey to accommodate site-specific language and activities, each survey followed the generic format outlined below.

Hello,

Thank you for taking the time to complete this survey. You are receiving this survey because your organization has been identified as a stakeholder in the State Innovation Model (SIM) Community and Clinical Care (C3) Initiative in [C3 site].

The primary goal of the C3 initiative is to encourage care coordination across medical, public health, and social service delivery systems.

The purpose of this survey is to collect information about the network of organizations involved in C3 related activities at the [C3 site], and you were identified as a representative of a participating organization.

The information collected in this survey will only be publicly available in aggregate form to make general observations about networks related to the C3 activities, implementation activities, and outcomes across all 7 C3 sites in the state. No information will be released publicly or among funders that identifies you as an individual, your organization, or C3 site.

The results of this survey and subsequent analysis will be disseminated to the [C3 site] for internal review and use.

Participation in this survey is voluntary.

Q1

This survey will ask about various collaborative activities your organization may contribute to as a participant in [C3 site] activities.

Examples of collaborative activities:

Advisory Role: *work together to guide the strategic direction of the C3*

Care Coordination: *Sends and/or receives client care coordination referrals across organizations*

Data Sharing: *Contribute to or have access to a shared database, share client data across organizations, share other data (surveys, focus groups, etc.) across organizations.*

Resource Sharing: *Contribute or receive resources through DMC C3 collaboration (e.g. monetary, training/ educational materials, space, staff time)*

Please select all (if any) organizations from the list below your organization works with for [C3 site] activities. Do not select your own organization.

- Organization 1
- Organization 2
- Organization 3

Q2

Please select the ways your organization collaborates with the organizations listed below. Please review the definitions of types of collaboration below and select all (if any) that apply.

Advisory Role: *work together to guide the strategic direction of the C3*

Care Coordination: *Sends and/or receives client care coordination referrals across organizations*

Data Sharing: *Contribute to or have access to a shared database, share client data across organizations, share other data (surveys, focus groups, etc.) across organizations.*

Resource Sharing: *Contribute or receive resources through DMC C3 collaboration (e.g. monetary, training/ educational materials, space, staff time).*

	Advisory Role	Care Coordination	Data Sharing	Resource Sharing
Organization 1	•	•	•	•
Organization 2	•	•	•	•
Organization 3	•	•	•	•

Q3

Please indicate the status of relationships/ level of trust between your organization and its collaborative partners in the [C3 site].

	New Relationship / Little Trust	Developing Relationship / Some Trust	Excellent relationship/ High Trust
Organization 1	•	•	•
Organization 2	•	•	•
Organization 3	•	•	•

Q4

Please select the option(s) that best describe collaborative relationships between your organization and other collaborating organizations involved in the [C3 site].

Please select all that apply.

	Collaboration preexisted SIM grant	Collaboration was stimulated by SIM participation	Collaboration is specific to only SIM project activities	Collaboration is expected to continue beyond SIM funding
Organization 1	•	•	•	•
Organization 2	•	•	•	•
Organization 3	•	•	•	•

Q5

Describe how, if at all, relationships between organizations participating in the [C3 site] have changed over the grant period (March 2016-present).

____

____

____

____

____
