# Supplementary material for: Applying network analysis to assess the development and sustainability of multi-sector coalitions
Source: PLoS One. 2022 Oct 18;17(10):e0276114. doi: 10.1371/journal.pone.0276114 (PMC9578585; doi:10.1371/journal.pone.0276114)
Supplement: Attachment — Submitted filename: Response to Reviewers Heeren et al.docx [file pone.0276114.s003.docx]

5. Review Comments to the Author

Please use the space provided to explain your answers to the questions above. You may also include additional comments for the author, including concerns about dual publication, research ethics, or publication ethics. (Please upload your review as an attachment if it exceeds 20,000 characters)

Reviewer #1: I enjoyed reading this paper. It is clearly and succinctly written and reports from an interesting study. I am not equipped to interrogate all of the methods but overall my sense is that the analyses are robust.

The study design matches its aims, and I particularly liked the attempt to measure relationships pre- and post the initiative. The paper reports some important findings, especially re the limitations of such funding approaches when relationship-building requires a longer view.

I think the exclusively quantitative design is a limitation as the tendency is to over-simplify complex social and organisational variables. Some qualitative insights might have added nuance here.

**Author response: Additional qualitative content added to provide additional context and illustrate motivation for network analysis methodology.**

My only suggestion for amending the paper is to elaborate a little on its contribution. The authors talk about ‘refining’ understanding but this could be tied to a prior assessment of limitations/gaps in the literature.

**Author response: A summary of contributions to documented gaps in literature have been added.**

Reviewer #2: This is a very interesting paper, but I would urge you all to go back and think about why you are doing this study, motivate with theory, what are you are capturing in the “networks” and justify your dyadic measures more.

There is no theoretical motivation for this research provided. Secondly, what are the networks you are trying to examine? How is each network bounded? Please explanation what the networks are, at least in theory. And what is the rationale for the dyad level measures? Please explain/give more motivation. Can we assume that most of the organizations have dyadic relationships? What about triads? Or clique structures?

**Author response:**

- **More explicit definition of network boundaries and membership added (formal membership in health coalitions).**
- **Underlying theory expounded upon (including structural holes, functional ties, and sustaining system change). Also referenced influencing implications from practical applications.**
- **Theoretical justification for examining dyad-level measures and network density were added (focus on referral network connectivity between organizations and overall were of primary interest related to research question)**
- **Triads were measured via network transitivity, but wasn’t expounded upon in discussion, as results were not integral to hypothesized outcomes**

Please explain the creation of the dyad-level covariate which is the absolute difference in closeness centrality, thus capturing pairwise similarity in centrality of network position. There are many centrality measures. Why is closeness centrality of theoretical or empirical importance herein?

**Author response:**

- **Added theoretical content justifying the applied function of closeness centrality in a referral network (e.g. indicates capacity to connect efficiently with each actor in the network)**

What was the extent of the missing data? Please elaborate on this.

**Author response: Table 1 revised to include % of missing edges**
