# Supplementary material for: Applying network analysis to assess the development and sustainability of multi-sector coalitions
Source: PLoS One. 2022 Oct 18;17(10):e0276114. doi: 10.1371/journal.pone.0276114 (PMC9578585; doi:10.1371/journal.pone.0276114)
Supplement: Attachment — Submitted filename: Response to Reviewers Heeren et al 8.26.22.docx [file pone.0276114.s004.docx]

6. Review Comments to the Author

Reviewer #3: This paper reports on an inter-organizational analysis relationship formed within the context of a state-wide granting mechanism in response to field reports on the challenges of creating and sustaining cross-sector collaborations. A few comments to improve the study.

☒The one glaring issue, which is certainly not amenable is the reliance on one time period to measures pre-SIM, during SIM, and projected sustainment. Although I am not immediately aware of any such studies I am certain there are other studies which have assessed networks at multiple time points at one time. I would encourage the authors to find such studies, reference, and see how they describe this limitation. Moreover, I wonder if the authors can explore any ways in which they can “validate” these reports.

The reviewer is correct, in both that this is a major limitation and one which due to study constraints we were unable to avoid. If the reviewer can point us to specific papers we would take them into consideration; however, we are unaware of any papers that investigate the potential ramifications of this study design. The rationale behind perceptions of predicted sustainment as a valid assessment is based in the Theory of Planned Behavior, which states that behavioral intention (in this case, sustaining a relationship) is associated with behavioral achievement (Azjen, 1991). Several prior studies investigating network change over time have called for additional research with longitudinal analysis including variables like trust, stage of relationship, collaborative task, and network size (Bryson, 2015; Provan 2012; Varda, 2008) The Provan 2012 study used longitudinal data, but noted this did not mitigate all the complexities of interpreting network change over time, “First, despite use of longitudinal data, we were unable to develop a full understanding of how network ties evolved. For example, more in-depth qualitative data might have enabled us to examine the impact of [Value Option, a behavioral health network] VO’s contract rules on the evolution of the network as a whole… we did not know how many relationships were already in place when the VO system first was established. Thus, while the VO system was new, many relationships among providers may have predated VO.”(p.373).

As a small step in this direction, we ran a simulation study, replicating the analysis for stimulated connections. In this study, we simulated 100 datasets that, in order to emulate recall error, we randomly deleted 10% of the pre-existing edges. We then, for each simulated dataset, computed the estimated odds ratio and confidence intervals. We found remarkably little variation in the estimates, as can be seen in the figure below, which shows the boxplots of the 100 simulations for the lower bound and upper bound of the confidence intervals as well as for the point estimate of the odds ratio. The original CI bounds and point estimates are superimposed in red asterisks. It is clear that even if there is poor recall in pre-existing connections between organizations, we are still able to obtain accurate inference.

Regarding the analysis of sustained edges, since we were unable to confirm whether or not a connection was in fact sustained, it might be better to interpret our results in terms of *intent* to sustain. We have changed the Limitations Section to reflect all these points.

☒Second, the authors make no mention of who specifically, within each organization, completed the survey. Presumably each organization has multiple hierarchical levels ranging from CEO, Director, program manager, staff, research assistant, etc. Who was responsible for providing this information can have implications for what they can report. Indeed, a CEO is likely to have quite a different perception of the working relationships than a program manager. This should be analyzed as it might be that the integrator organization consisting of health service agencies may have delegated reporting (survey completion) differently than other organizations.

Surveys were completed by the staff designated as primary contact(s) representing the organization in the ACH network. In cases in which multiple staff from a single organization were involved, survey responses were aggregated at the organization level. The survey sample was largely comprised of practitioner roles (as opposed to formal leadership roles in each organization), for example, primary care providers, local public health department staff, hospital clinic managers, behavioral health providers, school nurses, law enforcement staff (e.g. sheriff), community members, transportation providers, faith-based organization staff, pharmacists, health coaches, care coordinators, case managers, benefits program administrators (e.g. WIC), non-profit staff, local government representatives (e.g. city managers), community group leaders (e.g. local NAACP chapters), and program managers.

This phrase was added to line 136

Other stakeholders in Iowa’s ACHs included local representatives from hospitals, primary care providers, other healthcare providers (e.g., behavioral health, pharmacy, dental), insurers, community action organizations, governmental entities, and social service providers. Surveys were completed by the staff designated as primary contact(s) representing the organization in the ACH network. In cases in which multiple staff from a single organization were involved, survey responses were aggregated at the organization level. These representatives comprised the survey sample, which was largely practitioner perspectives (as opposed to formal leadership e.g., CEOs)

☒Line 139: A roster of all possible collaborators was roved to minimize recall error and strengthen the equity of reporting, a
“roved”? -> “provided

Line 139, previously corrected in November 2021 revision response

☒Line 153: For instance, some tasks require mutual trust and investment (e.g. data sharing) while execution of other activities (e.g. co-serving on an advisory board) are less intensive.(18)
Should this be “ … less interactive.”?

Line 153, changed to interactive

☒Line 179: you should cite Freeman, 1979 on closeness centrality and report which variant you use.

Freeman 1978 citation added to line 201, in addition to the phrase below, added to line 202

Closeness centrality was calculated as the sum of the inverse distances and measures how close..

This method of calculating closeness centrality is sometimes called “harmonic centrality,” a type of relative measure (as described in Freeman 1979). The specific variant employed was not described in the Freeman paper.

☒Lines233-234: as per comment 2 above, was there only one individual reporting for each organization?

No, see response to second comment RE sample

Lines262-263: “Contrary to the hypothesis that organizations with more similar centrality in the preexisting network were more likely to form stimulated edges with one another, there was a significant positive effect of the absolute difference in closeness centrality between the organizations (OR = 1.10, 95% CI = [1.04, 1.16]).”

☒Lines 264-265: “Meaning, organizations with dissimilar centralities (i.e. pairs comprised of one core and one periphery organization) were more likely to form connections.”
Make a visual inspection to be certain these are new core-periphery ties, just because they closeness centrality differences are associated with new ties does not necessarily imply that.

We acknowledge that our original language was too strong regarding stimulated edges between core and peripheral organizations. We have changed the text now to read,

“This statistically significant positive effect implies that organizations with more dissimilar centralities were more likely to form connections, such as a stimulated connection between a central hub and a peripheral organization.”

☒But I would think that organizations with high and similar closeness centrality scores would already be connected. Is this controlled for? At the very least they would have a shared partner thus more likely to become connected.

The analysis for stimulated connections was implicitly conditioning on the non-existence of the edge; that is, we are considering only dyads without a connecting edge, and seeing which factors predict a new connection being formed (if the reviewer is familiar with STERGM, this is akin to the “relational formation” half of the separable likelihood). In the analysis for sustained connections, we are implicitly conditioning on the edge existing (again, akin to the “relational dissolution” half of the STERGM likelihood), and we are also including as a covariate whether or not the edge was originally connected or newly stimulated.

☒It might be worth noting if these 7 sites were the 7 largest municipalities in Iowa (or not).

This phrase has been added to line 127 –

The seven ACH sites in the state were a mix of single county (urban) and multi-county (rural) sites, with the intention of developing models for future replication for both settings.

☒Lines156-157: “For each collaboration, respondents indicated: “little trust/new relationship,” “some trust/developing relationship,” or “high trust/strong relationship.”
You might note as a limitation that trust and duration of relationship are not synonymous.

In pilot survey testing, items were specific only to level of trust. Test respondents reported they were hesitant to rate collaborative relationships as lacking trust. While trust and duration of relationship are not synonymous, alternative language describing the stage of the relationship (i.e., new, developing, strong) was included to mitigate social desirability bias.

☒Reviewer #4: One suggestion I had is that in the “likely sustained” model, you might add an additional model that only includes the variables in the “stimulated” model. This would allow you to assess the degree to which these added variables (including strength of ties) might moderate the effects of your ACH structure variables. This potentially could provide additional insights.

We appreciate the suggestion from Reviewer 4. We have rerun the analysis for the sustained connections model, and our results are consistent with previously reported findings (see table below). Analysis was completed, results consistent with original findings, suggesting that there are not moderation effects. Because these results are consistent, i.e., there does not appear to be moderation effects to report on, for the sake of brevity we have opted for omitting this additional analysis in the manuscript.

|  | **Stimulated** | | |  | **Likely Sustained** | | |  | **Likely Sustained (reviewer #4)** | | |
| --- | --- | --- | --- | --- | --- | --- | --- | --- | --- | --- | --- |
| Variable | Odds Ratio (OR) | 95% confidence Interval (CI) for OR | P-value |  | Odds Ratio (OR) | 95% confidence Interval (CI) for OR | P-value |  | Odds Ratio (OR) | 95% confidence Interval (CI) for OR | P-value |
| ***Autoregressive measures*** |  |  |  |  |  |  |  |  |  |  |  |
| Sum of node degree | **1.157** | **(1.097, 1.221)** | **<0.01** |  | **1.062** | **(1.010, 1.116)** | **0.0192** |  | **1.097** | **(1.049, 1.147)** | **<0.01** |
| Similarity = abs diff in closeness cent | **1.092** | **(1.034, 1.154)** | **<0.01** |  | 1.054 | (0.954, 1.165) | 0.3038 |  | 1.041 | (0.951, 1.140) | 0.3843 |
| Same sector indicator | 1.259 | (0.767, 2.065) | 0.3625 |  | 1.440 | (0.873, 2.377) | 0.1531 |  | **1.744** | **(1.104, 2.754)** | **0.0171** |
| ***ACH structure*** |  |  |  |  |  |  |  |  |  |  |  |
| Integrator org | **2.715** | **(1.248, 5.909)** | **0.0118** |  | 1.217 | (0.557, 2.658) | 0.6222 |  | 1.626 | (0.805, 3.286) | 0.1756 |
| Both members of steering committee | **13.860** | **(6.305, 30.469)** | **<0.01** |  | 0.887 | (0.347, 2.269) | 0.8023 |  | 0.824 | (0.385, 1.765) | 0.6182 |
| One member of steering committee | **3.226** | **(1.999, 5.206)** | **<0.01** |  | 1.230 | (0.688, 2.200) | 0.4852 |  | 0.886 | (0.527, 1.490) | 0.6480 |
| Average strength of relationship |  |  |  |  | **1.881** | **(1.377, 2.569)** | **<0.01** |  |  |  |  |
| Stimulated by SIM |  |  |  |  | 0.695 | (0.411, 1.173) | 0.1729 |  |  |  |  |
| ***Collaboration types*** |  |  |  |  |  |  |  |  |  |  |  |
| Advisory |  |  |  |  | 1.534 | (0.859, 2.739) | 0.1486 |  |  |  |  |
| Care coordination |  |  |  |  | 1.264 | (0.763, 2.095) | 0.3630 |  |  |  |  |
| Data sharing |  |  |  |  | **2.129** | **(1.265, 3.585)** | **<0.01** |  |  |  |  |
| Resource sharing |  |  |  |  | **2.164** | **(1.321, 3.546)** | **<0.01** |  |  |  |  |
